# Maternal Age Trends and Patterns in Greece (1956–2023): A Nationwide Analysis

**DOI:** 10.7759/cureus.87827

**Published:** 2025-07-13

**Authors:** Nikolaos Vlachadis, Dimos Sioutis, Chrissi Christodoulaki, Nikolaos Machairiotis, Charalampos Theofanakis, Ioannis Tsakiridis, Themistoklis Dagklis, Konstantinos Louis, Periklis Panagopoulos

**Affiliations:** 1 Third Department of Obstetrics and Gynecology, National and Kapodistrian University of Athens, Medical School, Attiko University Hospital, Athens, GRC; 2 Third Department of Obstetrics and Gynecology, Aristotle University of Thessaloniki, Medical School, Hippokrateio Hospital, Thessaloniki, GRC

**Keywords:** advancing maternal age, fertility, greece, high-risk pregnancy, maternal age, time trends

## Abstract

Introduction: Advanced maternal age is associated with an increased risk of major pregnancy complications. This study aims to investigate long-term trends in maternal age at childbirth in Greece, providing a comprehensive analysis of the evolving distribution of live births by maternal age over time.

Materials and methods: We analyzed national data on 8,208,255 live births in Greece from 1956 to 2023, obtained from the Hellenic Statistical Authority and based on official birth certificate records. Maternal age at delivery was categorized into six groups: < 20, 20-24, 25-29, 30-34, 35-39, and ≥ 40 years. To assess the temporal shift in maternal age distribution, the annual proportion (%) of live births within each age group relative to the total live births per year was calculated, and trends were evaluated using joinpoint regression analysis. Annual percent change (APC) values were calculated along with 95% confidence intervals (95% CI).

Results: The mean maternal age at childbirth in Greece declined to a minimum of 26.1 years in 1983 and 1984, then increased substantially over the past four decades, reaching a historic high of 32.1 years in 2023. This upward trend was characterized by an APC of 0.7 (95% CI: 0.7 to 0.8) between 1984 and 2000, followed by a slightly slower APC of 0.4 (95% CI: 0.4 to 0.4) from 2000 to 2023. The modal maternal age group in Greece shifted from 20-24 years between 1969 and 1988 to 25-29 years during 1989-2004, while since 2005, the most common age group at childbirth has been 30-34 years. Since the 1980s, there has been a notable shift in the distribution of live births in Greece toward older maternal ages. This trend is marked by a significant decline in the proportion of births to women < 30 years of age and a substantial increase among those aged ≥ 30 years. The relative contribution of mothers aged 35-39 years rose from a historic low of 5.20% in 1980 to a record high of 26.87% in 2021, although it has stagnated since 2018. The proportion of neonates born to mothers aged ≥ 40 years increased dramatically with an APC of 2.5 (95% CI: 1.3 to 3.3) between 1984 and 1995, followed by a markedly steeper APC of 7.0 (95% CI: 6.8 to 7.3) from 1995 onward. The percentage for this age group increased from an all-time low of 1.23% in 1984 to a historic peak of 10.69% in 2023, representing one of the highest rates among developed countries.

Conclusion: Greece has experienced a significant rise in maternal age since the 1980s, driven by a relative decline in births among women < 30 years and a marked increase in the proportion of births to older mothers, particularly those aged ≥ 40 years. These demographic trends raise important concerns and pose substantial challenges to national perinatal health outcomes.

## Introduction

In recent decades, the trend of delayed childbearing and pregnancies at an advanced maternal age has become extremely common in developed countries. This postponement of childbirth has been associated with a significant decline in fertility rates, shaping the global demographic landscape, particularly in high-income countries [1,2]. The increasing fertility rates among women over the age of 35, and more recently over 40, have been influenced by a range of socioeconomic changes, including the rise in women's educational and socioeconomic status, the widespread use of contraceptive methods, and the growing availability and utilization of assisted reproductive technologies (ART) [3].

Delayed childbearing elevates the risk of infertility and is a key driver of declining fertility rates in developed societies [4,5]. Additionally, advanced maternal age is linked to serious pregnancy complications, including a higher likelihood of fetal loss, whether through first- or second-trimester miscarriage or third-trimester intrauterine death, as well as chromosomal and non-chromosomal abnormalities, often necessitating extensive and invasive prenatal screening [6-9]. Older pregnant women also face increased risks of hypertensive disorders (such as preeclampsia), gestational diabetes, preterm birth, fetal growth restriction, and delivering low birth weight neonates. Consequently, these factors heighten the likelihood of neonatal and infant mortality, morbidity, and admission to neonatal intensive care units. A major contributor to these adverse outcomes is the elevated rate of multiple pregnancies, largely due to medically assisted reproduction [6,7,10-13]. The resulting maternal and neonatal complications, intensified prenatal monitoring, higher cesarean delivery rates, and greater reliance on fertility treatments collectively impose significant healthcare costs [14,15].

In Greece, fertility rates began to plummet in the 1980s, driven by rapid sociocultural changes linked to Europeanization following the country’s accession to the European Union, as well as the modernization of women’s societal roles. Through successive waves of decline, birth rates reached their lowest historic levels after the onset of the economic crisis, and the country now finds itself entrenched in the low-fertility trap [16,17]. This study aims to analyze national trends in maternal age at childbirth in Greece from 1956 to 2023, with a focus on changes in mean maternal age and the distribution of live births across defined age groups. The objective is to provide insights into evolving demographic patterns and their potential implications for perinatal health.

## Materials and methods

Study population

This study used publicly available data obtained from the Hellenic Statistical Authority [18], derived from official birth certificate records. All live births registered in Greece between 1956 and 2023 were included, categorized by maternal age at the time of delivery (in completed years).

Inclusion and exclusion criteria

During the study period (1956-2023), a total of 8,215,729 live births were recorded in Greece. Of these, 7,474 (0.09%) were excluded due to unregistered maternal age data. Consequently, 8,208,255 live births were included in the final analysis.

Study parameters

The study initially examined the average age of mothers at the time of delivery. For the period 1975-2023, this was calculated as the arithmetic mean, i.e., the sum of maternal ages at delivery for all live births in the country divided by the total number of live births. For the period 1956-1974, the median maternal age was used as an approximation. Subsequently, for each year from 1956 to 2023, the percentage (%) of live births from mothers in each age group (< 20, 20-24, 25-29, 30-34, 35-39, and ≥ 40 years) was calculated relative to the total number of live births in that year.

Statistical analysis

Data analysis was carried out using Microsoft Excel 2010 (Microsoft Corporation, Redmond, Washington, USA). Trend analysis was performed with the Joinpoint Regression Program, version 5.2.0 (National Cancer Institute, USA). This software identifies joinpoints that mark specific time periods where statistically significant changes in trends occur. The analysis calculated the annual percent change (APC) for each segment defined by two joinpoints, with a maximum of seven segments allowed. Results are presented alongside 95% confidence intervals (95% CI), and statistical significance was determined using a p-value cutoff of < 0.05. Due to the extended duration of the examined period and in order to accurately capture significant subgroup variations, the trend analysis of the proportion of births per maternal age group was divided into two phases. The first phase encompassed the years from 1956 up to the historical peak for age groups under 30 years (<20, 20-24, and 25-29 years) or the historic lowest point for age groups ≥ 30 years (30-34, 35-39, and ≥40 years). The second phase extended from the end of the first period through to 2023. This methodological approach enabled the analysis of trends across a maximum of 14 segments throughout the entire period from 1956 to 2023. Recent trends in mean maternal age (2001-2023) were further examined using a linear regression model, with the slope coefficient (beta) and the coefficient of determination (R²) calculated.

## Results

**Figure 1:**
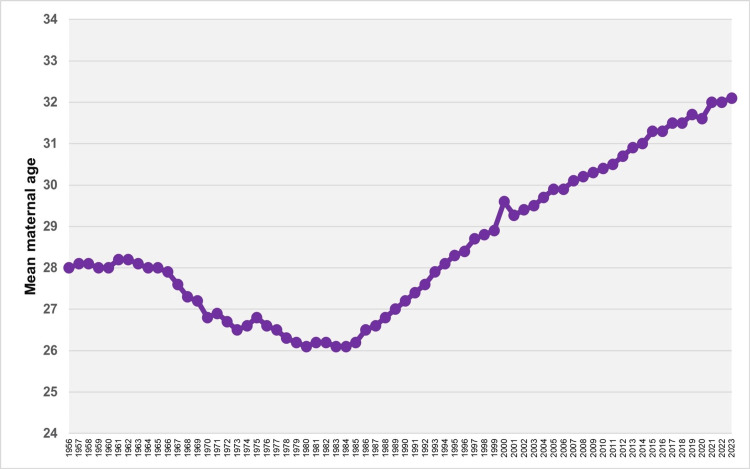
Mean maternal age in Greece, 1956-2023

**Figure 2:**
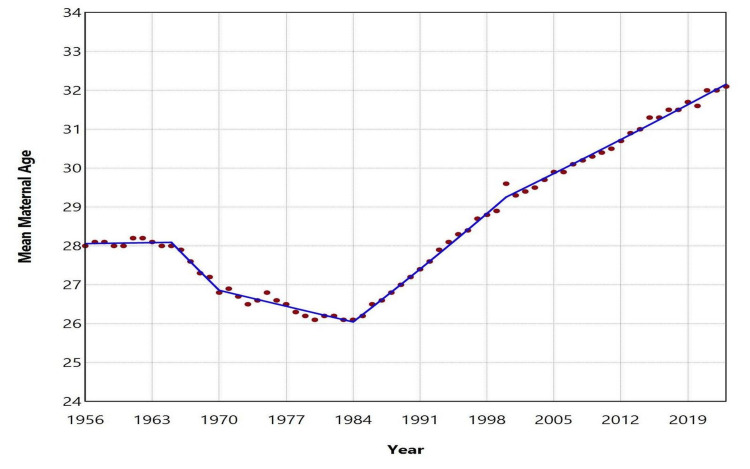
Trends in mean maternal age in Greece, 1956-2023

**Figure 3:**
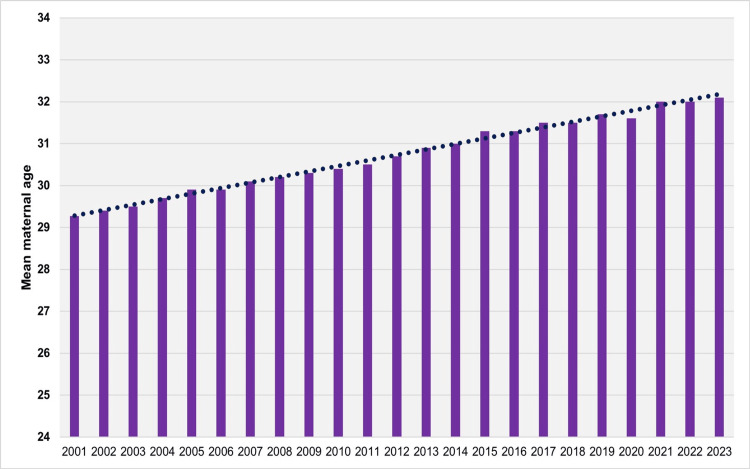
Regression line for mean maternal age in Greece, 2001-2023 Linear regression model: y = mean maternal age, x = year. Slope coefficient (β) = 0.13 (95% CI: 0.13 to 0.14, p < 0.001); coefficient of determination (R²) = 0.993

The mean maternal age at childbirth in Greece remained stable during 1956-1965 (p = 0.709), exhibiting minimal fluctuations at approximately 28.0 years, with a peak of 28.2 years observed in 1961 and 1962. A subsequent decline occurred over the next two decades: from 1965 to 1970, the APC was -0.9 (95% CI: -1.3 to -0.7, p < 0.001), and from 1970 to 1984, the APC was -0.2 (95% CI: -0.3 to -0.2, p < 0.001). This downward trend culminated in a recorded minimum of 26.1 years in both 1983 and 1984. Over the past four decades, the average maternal age has increased significantly, reaching a historic high of 32.1 years in 2023. From 1984 to 2000, the average annual increase was characterized by an APC of 0.7 (95% CI: 0.7 to 0.8, p < 0.001), followed by a more moderate rise from 2000 to 2023, with an APC of 0.4 (95% CI: 0.4 to 0.4, p < 0.001). A notable spike occurred in 2000, when the average maternal age rose sharply to 29.6 years from 28.9 years in 1999, before dipping slightly to 29.3 years in 2001. From 2001 to 2023, the trend in mean maternal age followed an almost perfectly linear trajectory (R² = 0.993), with a slope coefficient beta of 0.13 (95% CI: 0.13 to 0.14, p < 0.001). During this period, the only recorded decrease occurred in 2020 compared to 2019 (31.6 vs. 31.7 years), followed by an increase to 32.0 years in 2021 (Figures 1-3; Table 1).

**Table 1:** Trends in mean maternal age in Greece, 1956-2023 Trends were calculated using joinpoint regression analysis

Segment	Annual percent change	95% confidence interval	P-value
1956-1965	0.0	-0.1 to 0.1	0.709
1965-1970	-0.9	-1.3 to -0.7	< 0.001
1970-1984	-0.2	-0.3 to -0.2	< 0.001
1984-2000	0.7	0.7 to 0.8	< 0.001
2000-2023	0.4	0.4 to 0.4	< 0.001

**Figure 4:**
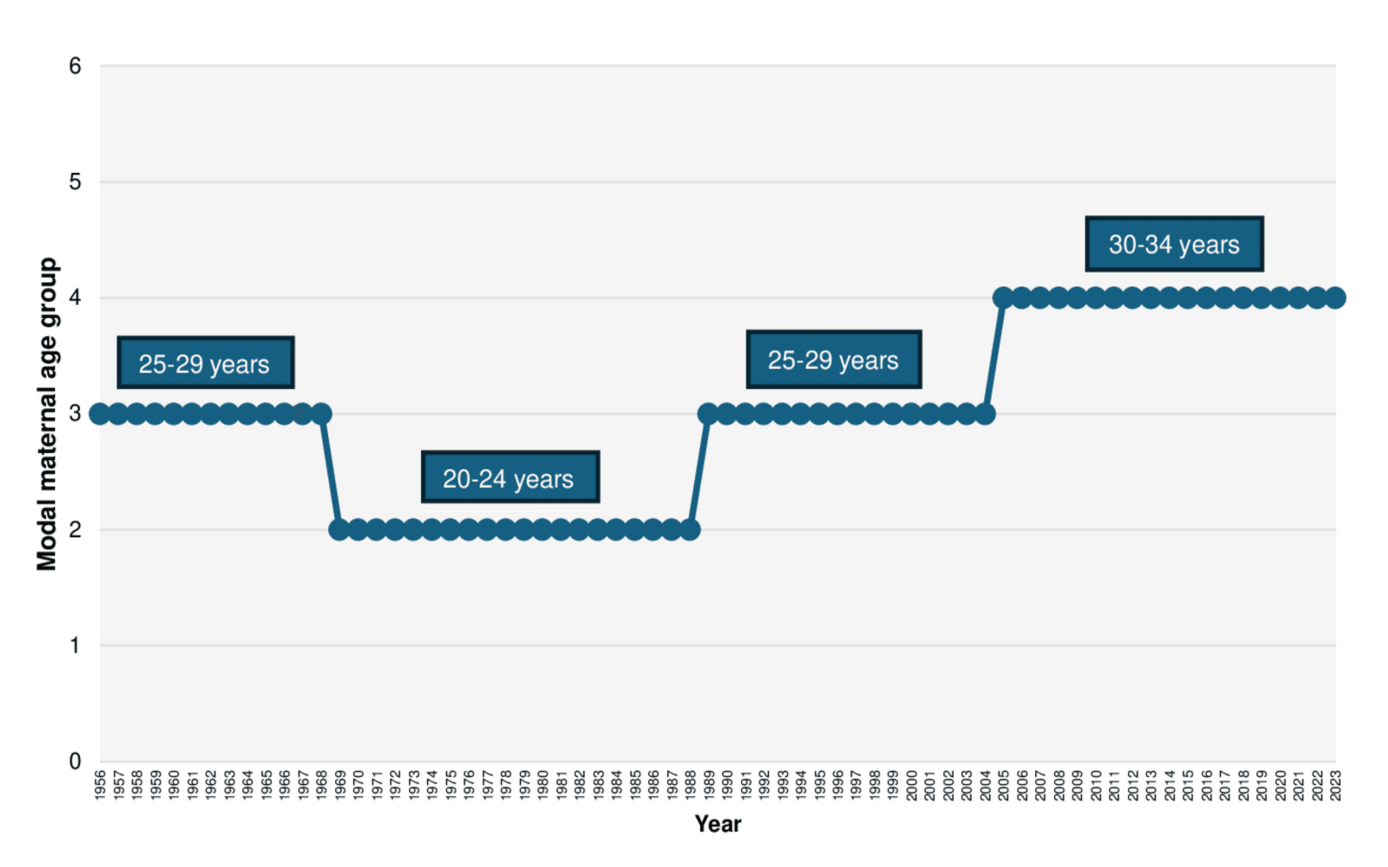
Modal maternal age group in Greece, 1956-2023 Maternal age groups: 1: < 20 years, 2: 20-24 years, 3: 25-29 years, 4: 30-34 years, 5: 35-39 years, 6: ≥ 40 years

The modal maternal age group in the Greek population was initially that of 25-29 years from 1956 to 1968. This was followed by a shift to the 20-24 age group, which dominated for approximately two decades from 1969 to 1988. The 25-29 age group once again became the most prevalent between 1989 and 2004. In the most recent period, from 2005 to 2023, the majority of neonates in Greece were born to mothers aged 30-34 years (Figure 4).

**Figure 5:**
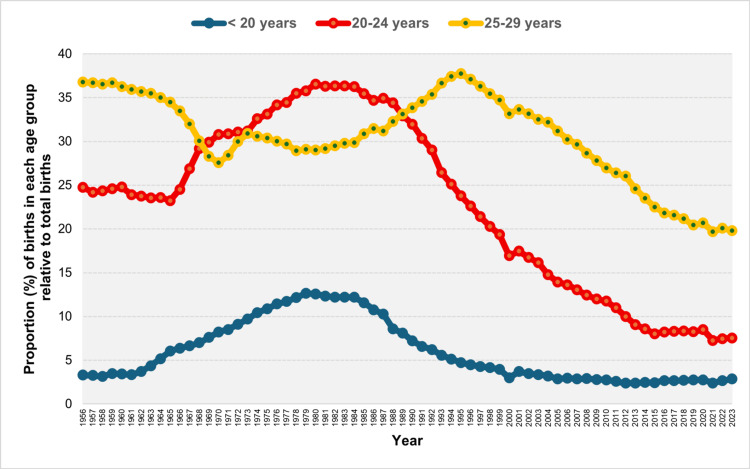
Distribution of live births by maternal age (%) for the < 30 years age subgroups (< 20, 20-24, and 25-29 years) in Greece, 1956-2023

**Figure 6:**
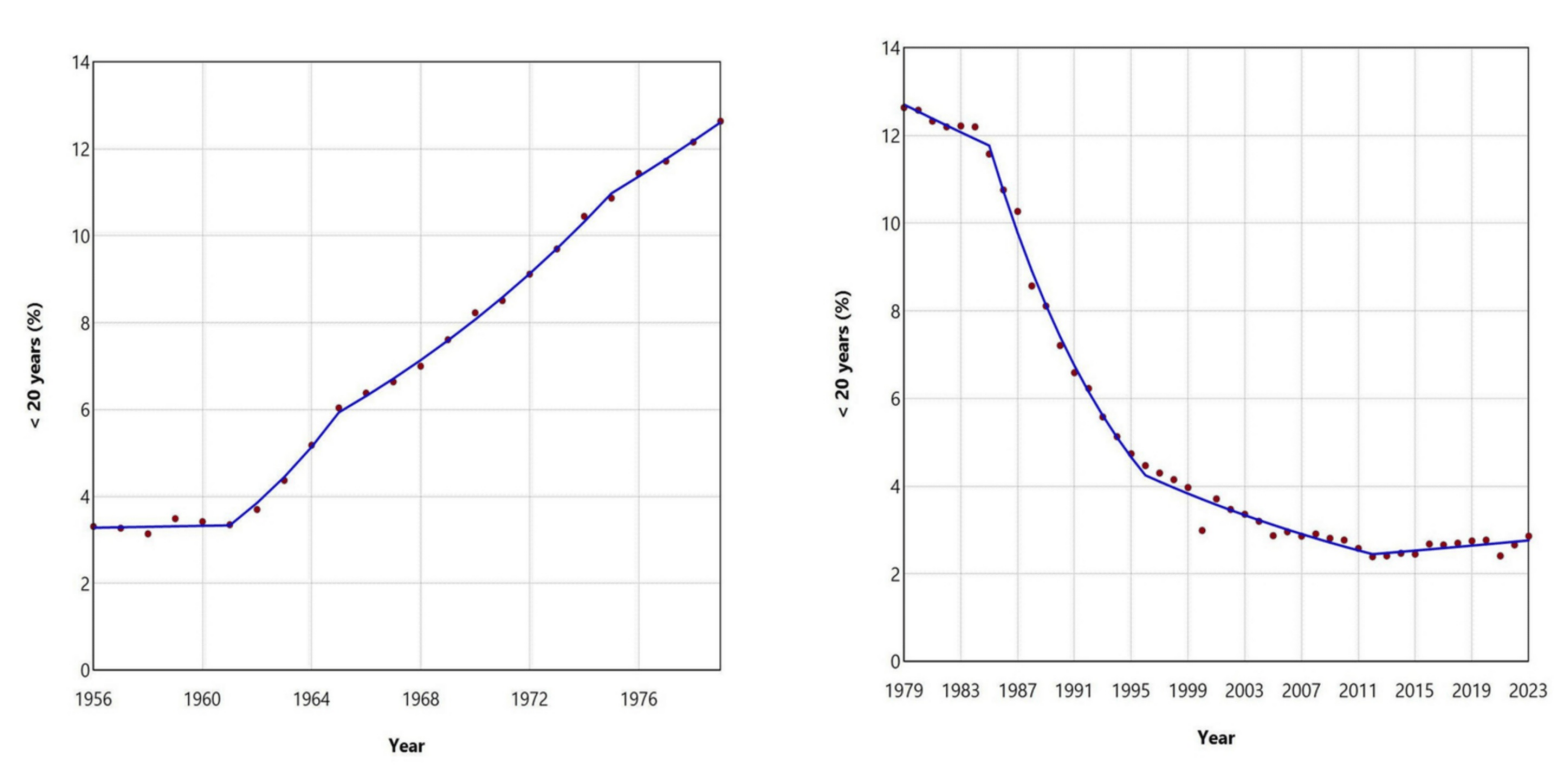
Trends in the percentage of live births from mothers aged < 20 years in Greece, 1956-1979 (left panel) and 1979-2023 (right panel)

The proportion of live births to women < 20 years in Greece increased from 3.14% in 1958 to a historic peak of 12.64% in 1979. This was followed by a steady decline, reaching an all-time low of 2.39% in 2012. In the most recent period (2012-2023), the percentage of births to mothers < 20 years old has shown a renewed upward trend, although this was marginally not statistically significant (APC = 1.1, 95% CI: -0.1 to 3.8). Notably, the proportion for this age group has risen consistently over the past three years: 2.41% in 2021, 2.66% in 2022, and 2.86% in 2023 (Figures 5, 6; Table 2).

**Table 2:** Trends in the percentage of live births from mothers aged < 30 years (< 20, 20-24, and 25-29 years) in Greece, 1956-2023 Trends were calculated using joinpoint regression analysis APC: annual percent change, 95% CI: 95% confidence interval

< 20 years			20-24 years			25-29 years		
Segment	APC	95% CI	Segment	APC	95% CI	Segment	APC	95% CI
1956-1961	0.3	-0.8 to 1.5	1956-1965	-0.7	-1.0 to -0.5	1956-1965	-0.7	-0.8 to -0.5
1961-1965	15.5	13.3 to 18.0	1965-1968	7.7	6.3 to 8.4	1965-1970	-4.8	-5.0 to -4.5
1965-1975	6.3	5.9 to 7.6	1968-1980	1.9	1.7 to 2.1	1970-1973	4.2	3.7 to 4.7
1975-1979	3.5	0.3 to 5.2	1980-1988	-0.8	-1.7 to 0.5	1973-1980	-1.1	-1.4 to -0.8
1979-1985	-1.3	-3.6 to 2.5	1988-2015	-5.1	-5.3 to -4.9	1980-1987	1.3	0.8 to 1.7
1985-1996	-8.8	-12.0 to -7.9	2015-2023	-1.4	-2.4 to -0.1	1987-1995	2.4	2.2 to 2.8
1996-2012	-3.4	-4.5 to -2.4	-	-	-	1995-2005	-1.8	-2.1 to -1.0
2012-2023	1.1	-0.1 to 3.8	-	-	-	2005-2012	-2.7	-3.5 to -2.0
-	-	-	-	-	-	2012-2016	-4.2	-5.2 to -1.3
-	-	-	-	-	-	2016-2023	-1.4	-2.0 to -0.6

**Figure 7:**
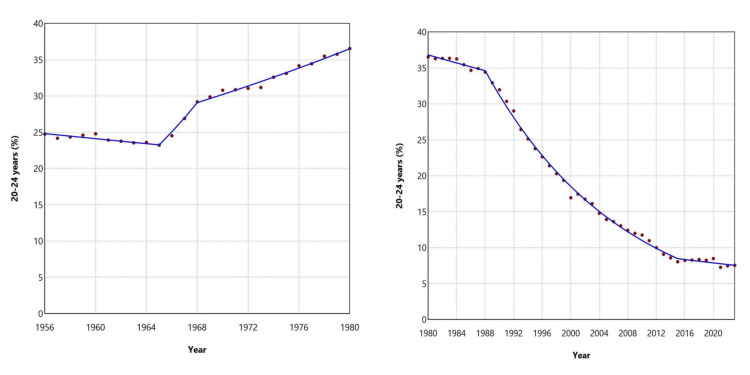
Trends in the percentage of live births from mothers aged 20-24 years in Greece, 1956-1980 (left panel) and 1980-2023 (right panel)

Following a decade of decline, the proportion of births to women aged 20-24 years rose from 23.23% in 1965 to a historic peak of 36.55% in 1980. Since then, the trend has reversed, with a particularly steep decline observed between 1988 and 2015, marked by an APC of -5.1 (95% CI: -5.3 to -4.9). In 2021, the proportion reached its lowest recorded level of 7.27% (Figures 5, 7; Table 2).

**Figure 8:**
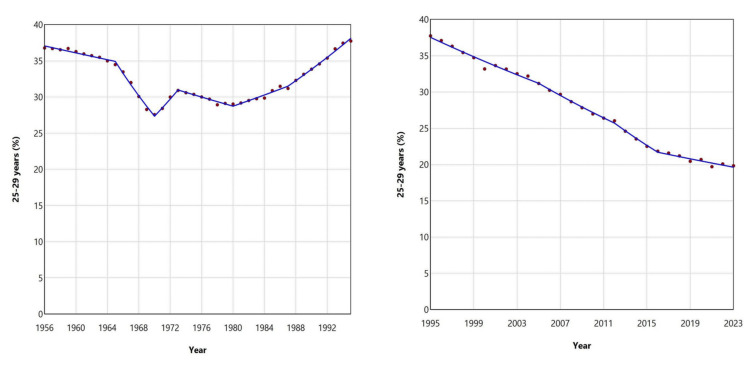
Trends in the percentage of live births from mothers aged 25-29 years in Greece, 1956-1995 (left panel) and 1995-2023 (right panel)

The 25-29 years age group experienced a decline in its share of births from 36.78% in 1956 to 27.57% in 1970, followed by a substantial increase, reaching an all-time high of 37.74% in 1995. Over the past three decades, however, the proportion of births to mothers aged 25-29 years has continuously declined, reaching a historic low of 19.70% in 2021 (Figures 5, 8; Table 2).

**Figure 9:**
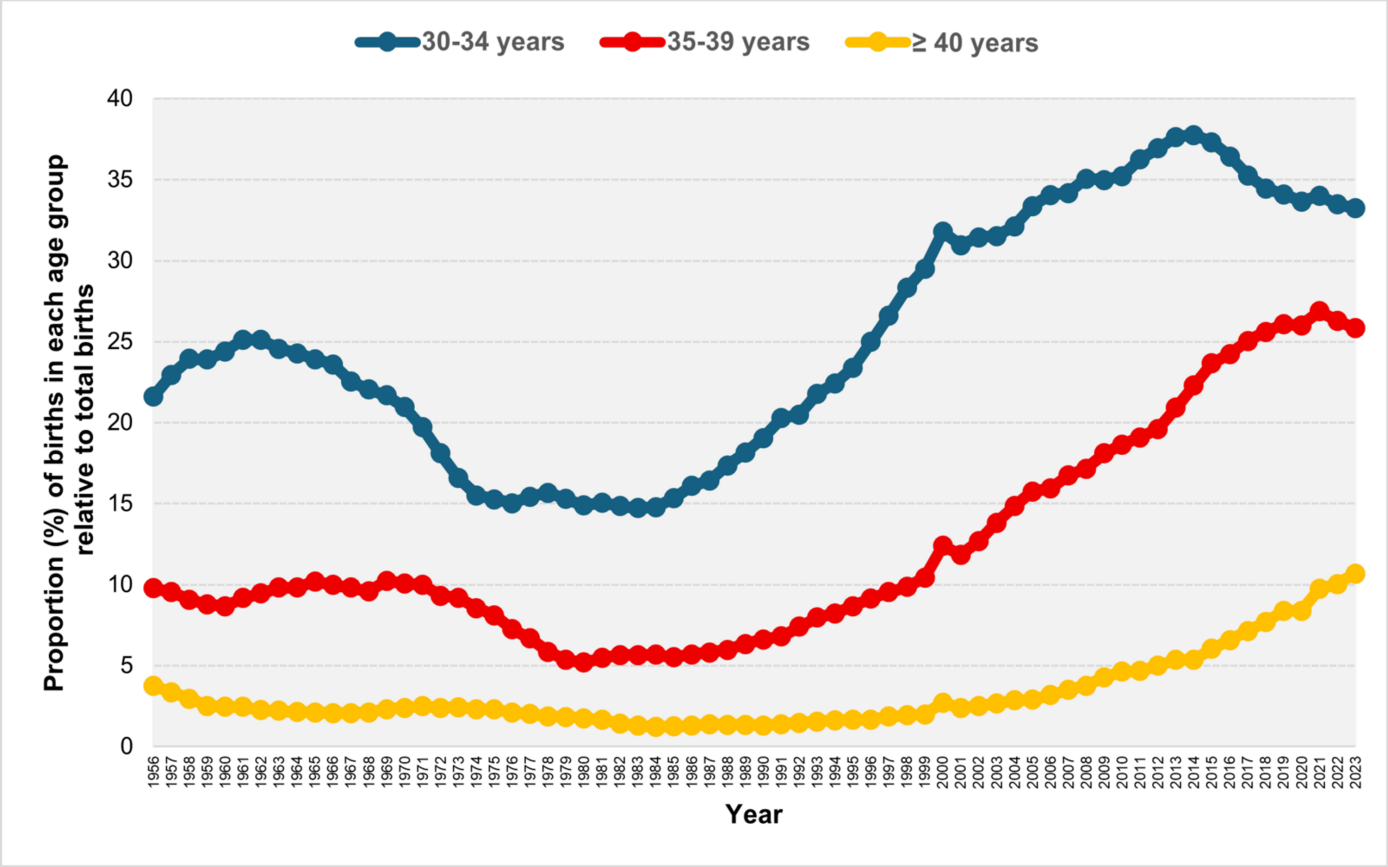
Distribution of live births by maternal age (%) for the ≥ 30 years age subgroups (30-34, 35-39, and ≥ 40 years) in Greece, 1956-2023

**Figure 10:**
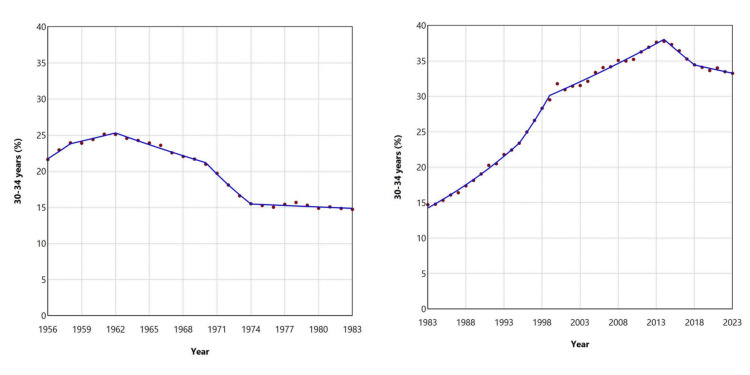
Trends in the percentage of live births from mothers aged 30-34 years in Greece, 1956-1983 (left panel) and 1983-2023 (right panel)

After a brief increase, the relative contribution of the 30-34 age group to total live births in Greece declined from 25.13% in 1961 to an all-time low of 14.72% in 1983. This was followed by a steady upward trend, culminating in a historic high of 37.76% in 2014. During the most recent period (2014-2023), the proportion decreased to 33.25% in 2023; however, no statistically significant trend was observed (Figures 9, 10; Table 3).

**Table 3:** Trends in the percentage of live births from mothers aged ≥ 30 years (30-34, 35-39, and ≥ 40 years) in Greece, 1956-2023 Trends were calculated using joinpoint regression analysis APC: annual percent change, 95% CI: 95% confidence interval

30-34 years			35-39 years			≥ 40 years		
Segment	APC	95% CI	Segment	APC	95% CI	Segment	APC	95% CI
1956-1958	4.7	2.2 to 6.8	1956-1959	-4.1	-7.9 to -1.2	1956-1959	-12.5	-17.0 to -9.8
1958-1962	1.5	-3.1 to -2.1	1959-1965	2.7	1.5 to 5.9	1959-1966	-3.2	-4.5 to -1.8
1962-1970	-2.2	-8.0 to -1.5	1965-1973	-0.9	-2.1 to -0.1	1966-1972	3.7	2.4 to 6.0
1970-1974	-7.6	-8.5 to 0.0	1973-1980	-8.6	-9.6 to -7.8	1972-1980	-4.5	-5.2 to -3.5
1974-1983	-0.4	-1.0 to 0.5	1980-1988	1.1	0.2 to 1.9	1980-1984	-9.0	-11.8 to -7.4
1983-1995	4.2	3.9 to 4.5	1988-2005	5.8	5.5 to 6.2	1984-1995	2.5	1.3 to 3.3
1995-1999	6.5	4.2 to 7.8	2005-2018	4.2	3.6 to 4.7	1995-2023	7.0	6.8 to 7.3
1999-2014	1.6	1.4 to 6.2	2018-2023	0.2	-2.1 to 1.6	-	-	-
2014-2018	-2.5	-3.7 to 1.7	-	-	-	-	-	-
2018-2023	-0.7	-1.5 to 0.9	-	-	-	-	-	-

**Figure 11:**
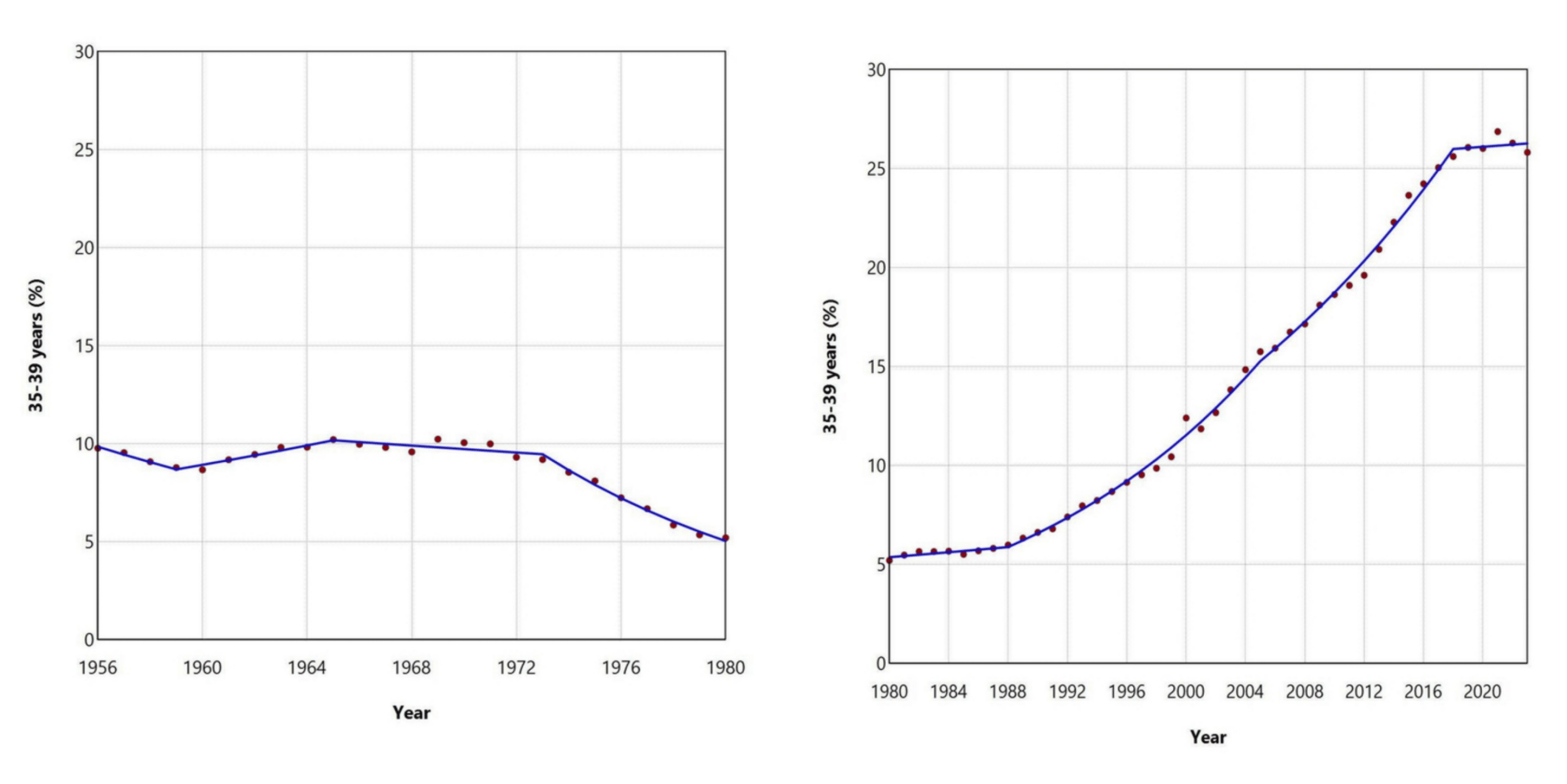
Trends in the percentage of live births from mothers aged 35-39 years in Greece, 1956-1980 (left panel) and 1980-2023 (right panel)

The relative contribution of mothers aged 35-39 years to the total number of live births in Greece declined from 10.21% in 1965 to a historic low of 5.20% in 1980. Subsequently, it rose sharply, by a factor of 5.2, to a record high of 26.87% in 2021. The upward trend exhibited distinct phases: a moderate APC of 1.1 (95% CI: 0.2 to 1.9) between 1980-1988, followed by faster growth from 1988 to 2018 (1988-2005: APC = 5.8, 95% CI: 5.5 to 6.2, 2005-2018: APC = 4.2, 95% CI: 3.6 to 4.7), whereas it stabilized post-2018 (Figures 9, 11; Table 3).

**Figure 12:**
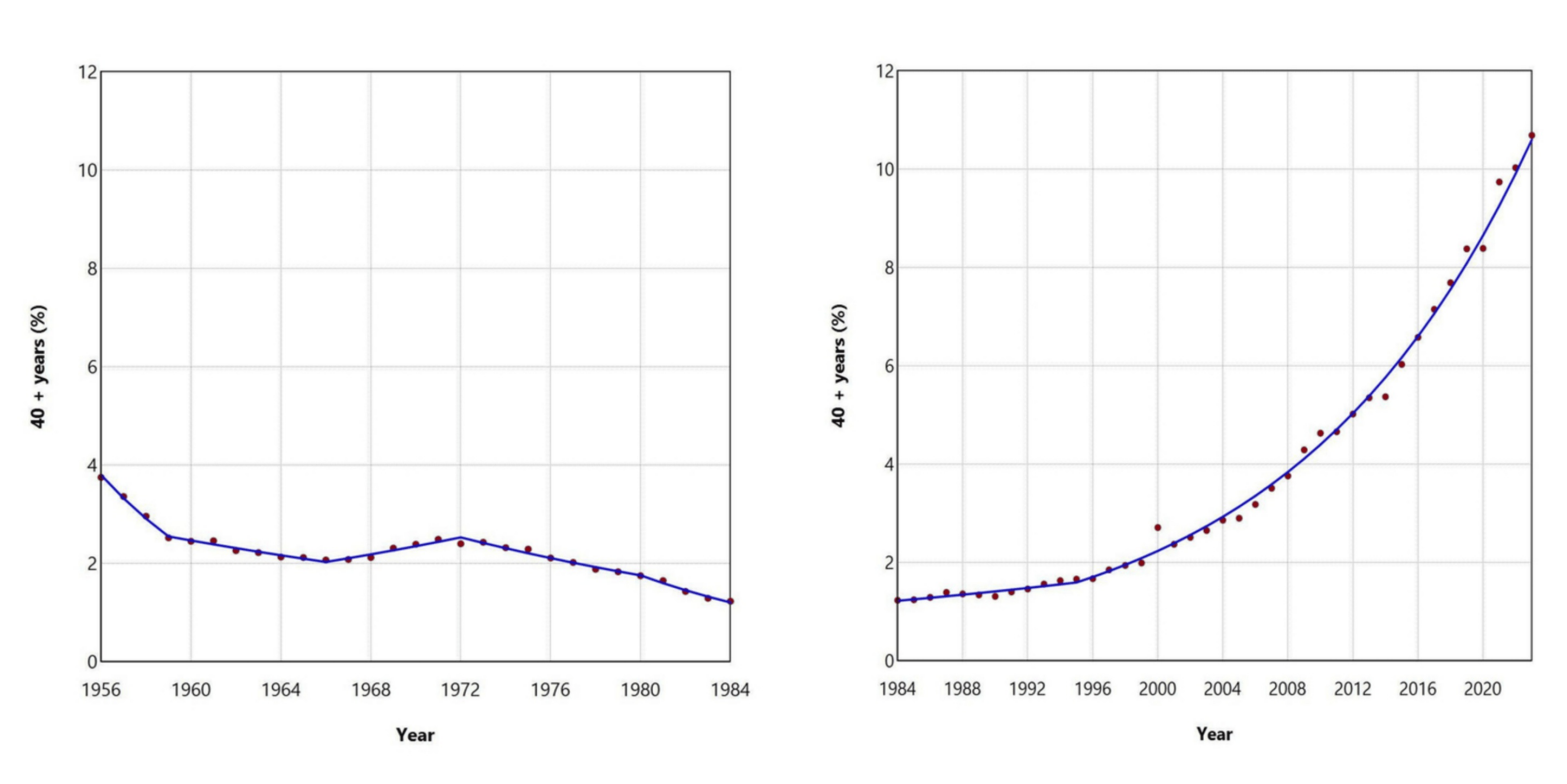
Trends in the percentage of live births from mothers aged ≥ 40 years in Greece, 1956-1984 (left panel) and 1984-2023 (right panel)

The percentage of neonates born to mothers aged ≥ 40 years experienced an initial period of decline, from 3.75% in 1956 to a historic low of 1.23% in 1984, showing a consistent downward trend except for the period between 1966 and 1972. Since 1984, this percentage has increased 8.7fold, to 10.69% in 2023, with an APC of 2.5 (95% CI: 1.3 to 3.3) between 1984 and 1995, and a faster APC of 7.0 (95% CI: 6.8 to 7.3) from 1995 to 2023 (Figures 9, 12; Table 3).

In the most recent period, two noteworthy years stand out: 2000, marking the turn of the millennium, and 2021, the year following the COVID-19 pandemic-related lockdowns. In 2000, compared to 1999, there was a decline in birth percentage among younger age groups (< 30 years) and an increase among older age groups (≥ 30 years). This shift was especially pronounced in the extreme age categories: for women < 20 years, the proportion decreased from 3.97% in 1999 to 2.99% in 2000, a decline of 25%. For the 20-24 years age group, it declined from 19.36% to 16.95%, a reduction of 12%. Conversely, for women aged 35-39 years, the percentage rose from 10.44% to 12.40%, an increase of 19%, and for women aged ≥ 40 years, it rose from 1.99% to 2.71%, an increase of 36%. In contrast, in 2021, compared to 2020, there was an increase in the contribution of births from women < 30 years, while the proportion among women aged ≥ 30 years decreased. The trend was also especially notable in the extreme age groups: for women < 20 years, the percentage decreased from 2.77% in 2020 to 2.41% in 2021, a decline of 13%. In the 20-24 age group, it fell from 8.49% to 7.27%, a decrease of 14%. Conversely, the proportion of births from women aged ≥ 40 years increased by 16%, rising from 8.39% in 2020 to 9.74% in 2021 (Figures 5, 9).

## Discussion

This study examined in detail the time trends in maternal age at childbirth among women in Greece over a period of nearly seven decades. The study period can be divided into two main phases, with the 1980s serving as a key turning point. The findings showed that the mean maternal age declined until approximately the mid-1980s and has steadily increased since then. This shift was accompanied by notable changes in the age distribution of births, particularly among the youngest and oldest maternal age groups. Initially, the proportion of births rose among younger women and declined among older age cohorts. However, from the 1980s onward, these trends reversed, culminating in a substantial increase in birth rates among women of advanced maternal age (≥ 35 years).

Following an initial decade of stability, the mean maternal age declined over the course of approximately 20 years, reaching its lowest recorded value of 26.1 years in 1983 and 1984. Since then, it has increased steadily, with an average annual rise of 0.7% during the period 1984-2000 and 0.4% per year since 2000, eventually reaching its highest historic level of 32.1 years in 2023. Excluding the sharp increase observed in 2000, the mean maternal age at childbirth in Greece rose on average by 0.13 years per year between 2001 and 2023, following an almost perfectly linear trend. Subsequently, trends in the most common maternal age group in Greece were analyzed. The majority of live births were to mothers aged 25-29 years until 1968, followed by a shift to the younger age group of 20-24 years, which remained dominant until 1988. The 25-29 age group then became the most common again until the mid-2000s. Since 2005, the most prevalent maternal age group has shifted to 30-34 years.

In the final phase of the study, the long-term trends in the distribution of births by maternal age group were analyzed. The proportion of births to mothers under the age of 25 increased during the first part of the period under review, reaching historic peaks in 1979 and 1980 for adolescent mothers (< 20 years) and women aged 20-24 years, respectively. Among adolescent mothers, the increase was fourfold. Trends were more complex for the 25-29 years age group, which peaked later, in 1995. In contrast, the share of births to women aged ≥ 30 declined, reaching historic low levels in 1980 for the 35-39 years age group, in 1983 for the 30-34 years group, and in 1984 for mothers aged ≥ 40 years.

These trends reversed during the second phase, from the 1980s to the present. The contribution of younger age groups declined to historic lows in 2021 for the 20-24 and 25-29 groups, while adolescent motherhood reached its lowest point earlier, in 2012. Conversely, the share of births to women aged 35-39 years and ≥ 40 years peaked in 2021 and 2023, respectively, while the 30-34 years group reached its maximum slightly earlier, in 2014. The increase in the share of births among older mothers was substantial: more than fivefold for women aged 35-39 years, and nearly ninefold for those aged ≥ 40 years.

The analysis of recent trends indicates a statistically significant downward trajectory in the proportion of births among women aged 20-29 years, while the trends observed for the <20 and 30-39 years age groups are not statistically significant. Notably, emerging upward trends at both extremes of the maternal age spectrum raise concern. In particular, the adolescent age group has exhibited an upward trend since 2013, which is marginally statistically significant, with a consistent year-on-year increase observed between 2021 and 2023. The only age group that continues to demonstrate a statistically significant upward trend is that of women aged ≥ 40 years, whose share of total births has been increasing at a sustained and substantial annual rate of approximately 7% since 1995.

A woman's natural fertility decreases dramatically with age. This decline becomes particularly pronounced after the age of 30, and by the age of 40, the majority of pregnancies are achieved through ART therapies [1,4]. In Greece, unlike many other European countries, there was no post-war baby boom in the 1950s. The decline in fertility began later (during the 1980s) compared to Western Europe, where it started in the 1970s. National birth trends in Greece closely mirror changes in maternal age: birth rates remained relatively stable from the 1950s through the 1970s, with only a slight overall decrease. During this period, births among younger women were maintained or even increased. This situation changed completely during the 1980s, marked by radical changes in Greek society following the country's accession to the European Union. Delays in having children, driven by shifts in the social roles of women and a lack of supportive family policies, led to a dramatic decline in birth rates nationwide [16,17]. At the same time, there was a noticeable increase in births among women aged ≥ 35 years, with the oldest age group (≥ 40 years) becoming the fastest-growing segment of births over the past approximately three decades.

A study comparing changes in average maternal age between 1990 and 2020 found an increase of 3.3-3.4 years in Spain, Italy, and Norway; 2.7-2.9 years in Denmark, Sweden, and the United Kingdom; and a smaller rise of 2.3-2.5 years in France, the United States, and Finland [19]. In contrast, Greece recorded a significantly larger increase of 4.4 years, from 27.2 years in 1990 to 31.6 years in 2020. In the United States in 2023, 16.8% of live births were to mothers aged 35-39 years and 4.1% to those aged ≥ 40 years [20], compared to 25.8% and 10.7%, respectively, in Greece.

The longitudinal study of the distribution of births by maternal age in Greece from 1956 to 2023 revealed two notable and abrupt shifts. The first occurred in the year 2000, when, compared to 1999, there was a sharp decline in the proportion of births among younger mothers < 30 years, accompanied by a corresponding increase in births among women aged ≥ 30 years. These changes were most pronounced at the age extremes. This shift may be attributed to cultural beliefs surrounding the turn of the millennium, which were particularly influential among younger mothers, typically of lower educational attainment. The second, inverse shift was observed in 2021 compared to 2020, likely reflecting a “rebound” effect following the suspension of fertility treatments during the COVID-19 pandemic [16].

The skyrocketing trend in birth proportions among women aged ≥ 40 years in recent decades in Greece is closely linked to the extensive reliance on ART therapies. The most recent data from the European Society of Human Reproduction and Embryology (ESHRE) for the years 2018 and 2019 highlighted that Greece had the highest proportion of women aged 40 and over among patients undergoing in vitro fertilization (IVF) [21]. Specifically, in 2019, more than half of all aspirations in Greece (54.2%) were performed in women aged ≥ 40 years, a figure nearly three times higher than the average across 30 countries, which stood at just 19.0% [21].

The analysis of live birth data by maternal age in Greece revealed concerning trends related to the increasing average age of motherhood, marked by a significant rise in the proportion of births to women ≥ 35 years. The most notable increase was observed among women in the oldest age group, those aged ≥ 40 years. In 2021, Greece had the sixth-highest average maternal age at childbirth among countries of the Organisation for Economic Co-operation and Development (OECD) [22], and by 2023, it ranked third in the European Union in the proportion of births to women aged ≥ 40 years, behind only Ireland and Spain [23]. A previous study examined maternal age trends in Greece from 1980 to 2008, noting that the shift toward older motherhood was more pronounced in the capital, Athens, compared to other regions of the country [15]. Another publication reported that, between 1990 and 2010, the increase in the contribution to live births in Greece was progressively greater with advancing maternal age among women ≥ 30 years [24].

The unfavorable trends in age at childbirth in Greece carry significant public health implications. Advanced maternal age is associated with a range of serious pregnancy complications, including an increased incidence of congenital anomalies in the fetus and pregnancy loss, as well as a higher risk of major morbidity-related complications for both the mother and the newborn [7,10,25]. Poor perinatal outcomes are associated with pre-existing medical conditions in older women prior to conception but also with age-related pathophysiological alterations in the endocrine and reproductive systems, as well as with placental insufficiency [26]. Advanced maternal age has been identified as a significant factor contributing to the deterioration of preterm and low birth weight rates, which constitute critical public health challenges in Greece [13,27,28]. Further investigation is warranted to elucidate the precise impact of advanced maternal age on the worsening perinatal rates within the Greek population.

Our findings highlight a marked shift in the distribution of births toward older maternal ages beginning in the 1980s. The most prominent trend in recent decades has been the substantial increase in the proportion of births to women aged ≥ 40 years, a pattern largely attributed to the widespread use of ART. This study provides a comprehensive analysis of maternal age birth patterns in Greece, with the distinct advantage of covering nearly seven decades and including data from over 8 million live births. These findings provide valuable insights and establish a robust foundation for future epidemiological research. One of the key strengths of this study is the reliability of the data, which is derived from official birth certificate records. However, several limitations should be acknowledged. This analysis focused on the distribution of births by maternal age group, as these indicators are critical for shaping perinatal health metrics. For instance, key indicators such as the preterm birth rate, which is calculated as the proportion of preterm births among all live births, are determined by the percentage of births in each maternal age group, rather than by their absolute numbers. Nonetheless, further analyses incorporating age-specific population data and calculating age-specific fertility rates could offer a more comprehensive understanding of the impact of maternal age on the demographic birth trends in Greece. Moreover, the study is purely descriptive and does not analyze causal factors influencing the shift in maternal age (e.g., economic trends, fertility treatments, policy changes). Individual-level sociodemographic variables, such as educational attainment, socioeconomic and marital status, were not available, thereby restricting insights into how these factors influence trends in maternal age. The available data did not distinguish between birth orders to present the differences between primiparous and higher-order mothers. The study also relied on aggregated, national-level data, which limited the ability to examine regional variations, urban-rural differences, or distinctions between Greek nationals and immigrant populations. Future research should seek to incorporate these dimensions to better elucidate the underlying social, economic, and demographic drivers of delayed childbearing in Greece.

## Conclusions

This study demonstrates an upward trend in average maternal age in Greece over recent decades, characterized by a significant shift toward older motherhood and a marked increase in births to women aged ≥ 40 years. Such demographic changes highlight critical public health challenges, including obstacles to reversing declining fertility rates, an increased prevalence of high-risk pregnancies, and a consequent deterioration in perinatal outcomes, along with rising healthcare costs. As advances in ART continue to expand, there is a growing need for ongoing epidemiological monitoring and further research to guide evidence-based public health policies and clinical practice.
